# Mitigating Post-Subarachnoid Hemorrhage Complications: Anti-Inflammatory and Anti-Apoptotic Effects of Anakinra in an Experimental Study

**DOI:** 10.3390/jcm14041253

**Published:** 2025-02-14

**Authors:** Güven Kılıç, Berk Enes Engin, Amir Halabi, Cengiz Tuncer, Mehmet Ali Sungur, Merve Alpay, Adem Kurtuluş, Hakan Soylu, Ali Gök, Ömer Polat

**Affiliations:** 1 Department of Neurosurgery, Faculty of Medicine, Duzce University, 81620 Duzce, Türkiye; drberkengin@gmail.com (B.E.E.); amrhalabi90@gmail.com (A.H.); cengiztuncer@gmail.com (C.T.); dr.ademkurtulus@gmail.com (A.K.); 2 Department of Biostatistics, Faculty of Medicine, Duzce University, 81620 Duzce, Türkiye; malisungur@yahoo.com; 3 Department of Biochemistry and Molecular Biology, Faculty of Medicine, Duzce University, 81620 Duzce, Türkiye; mervealpay@duzce.edu.tr; 4 Department of Histology and Embryology, Faculty of Medicine, Duzce University, 81620 Duzce, Türkiye; hknsyl85@gmail.com; 5 Experimental Animals Application and Research Center, Duzce University, 81620 Duzce, Türkiye; aligok@duzce.edu.tr; 6 Department of Neurosurgery, Cagsu Hospital, 81600 Bolu, Türkiye; polatnrs@gmail.com

**Keywords:** anakinra, apoptosis, inflammation, interleukin-1 receptor antagonist, subarachnoid hemorrhage, vasospasm

## Abstract

**Background**: Subarachnoid hemorrhage (SAH) is a severe neurological condition with high mortality and morbidity rates, often exacerbated by secondary complications such as inflammation, cerebral vasospasm, and apoptosis. Proinflammatory cytokines, including interleukin-1 (IL-1), tumor necrosis factor-alpha (TNF-α), and interleukin-6 (IL-6), play critical roles in these pathological processes. Anakinra, an IL-1 receptor antagonist, has demonstrated significant anti-inflammatory effects in various disease models. This study aimed to evaluate the efficacy of anakinra in mitigating inflammation, vasospasm, and apoptosis in an experimental rat model of SAH. **Methods**: Thirty-two male Sprague Dawley rats were divided into four groups: Control (healthy), SAH (no treatment), Saline (0.2 mL saline subcutaneously), and Anakinra (50 mg/kg subcutaneously, twice daily). Proinflammatory markers (CRP, TNF-α, IL-1, IL-6, and fibrinogen) were measured in serum and cerebrospinal fluid (CSF) at 3, 7, and 10 days post-SAH. Basilar artery diameter was evaluated histopathologically, and Caspase-3 expression was assessed immunohistochemically to determine apoptotic activity. **Results**: SAH significantly increased levels of CRP, TNF-α, IL-1, IL-6, and fibrinogen in both serum and CSF, reduced basilar artery diameter, and elevated Caspase-3 expression compared to the Control group. Saline treatment provided limited improvements, with inflammatory markers and histopathological parameters remaining elevated. Anakinra treatment significantly reduced inflammatory markers, restored basilar artery diameter, and lowered Caspase-3 expression, highlighting its efficacy in mitigating inflammation, vasospasm, and apoptosis. **Conclusions**: Anakinra effectively suppresses inflammation, alleviates cerebral vasospasm, and inhibits apoptosis in an experimental model of SAH. These findings suggest its potential as a therapeutic agent for managing SAH and its complications. Further research is needed to explore its clinical applicability and long-term effects.

## 1. Introduction

Subarachnoid hemorrhage (SAH) is a clinical condition typically caused by the rupture of an intracranial aneurysm and characterized by high morbidity and mortality rates. Early diagnosis and intervention are critical in the management of SAH; however, complications that develop post-SAH remain major challenges in clinical practice. Among these, cerebral vasospasm is the most significant, arising from the accumulation of blood in the subarachnoid space, leading to secondary ischemia and poor neurological outcomes [1,2]. The pathophysiology of vasospasm is highly complex, involving mechanisms such as free radicals, inflammatory cytokines, endothelial dysfunction, and apoptosis [3].

Inflammation plays a central role in the development of vasospasm. Elevated levels of proinflammatory cytokines, including interleukin-1 (IL-1), tumor necrosis factor-alpha (TNF-α), and interleukin-6 (IL-6), have been observed following SAH. These cytokines disrupt the function of cerebral vascular endothelial cells, exacerbating the inflammatory response [4,5,6] (Yılmaz, 2010; Aydogmus et al., 2019). Controlling post-SAH inflammation is a primary target for preventing vasospasm and improving neurological outcomes.

In this context, IL-1 receptor antagonists have gained attention. Anakinra, a biological agent that inhibits the biological activities of IL-1, has demonstrated effectiveness in modulating inflammation and has been proven to be effective in the treatment of inflammatory diseases. Previous studies have reported that anakinra, particularly in conditions such as rheumatoid arthritis, can reduce inflammation and provide neuroprotective effects [7,8]. However, studies on the effects of anakinra on post-SAH inflammation and vasospasm remain limited, necessitating further preclinical studies to understand its therapeutic potential. In addition, anakinra has been previously investigated in both clinical and experimental SAH models, demonstrating promising anti-inflammatory effects. However, its impact on cerebral vasospasm and apoptosis remains incompletely understood, necessitating further experimental validation [4,7].

The use of saline as a control group is a common methodology in the literature for evaluating treatment efficacy. Saline is frequently utilized as a neutral reference, with minimal impact on the inflammatory response, representing the natural progression of biological processes [9,10]. These characteristics make the saline group critical for meaningful comparisons between treatment and control groups, providing a baseline for evaluating the biological effects of therapeutic agents.

This study investigates the effects of inflammation and cerebral vasospasm in an experimental SAH model using Control, SAH, Saline, and Anakinra groups. By evaluating serum and cerebrospinal fluid (CSF) biomarkers alongside histological parameters, the neutral effects of saline and the modulatory effects of anakinra on inflammation and vascular dysfunction are compared. The findings of this study are expected to provide significant insights into the biological effects of post-SAH inflammation and contribute to the development of novel therapeutic approaches.

## 2. Materials and Methods

### 2.1. Animal Groups and Experimental Design

A total of 32 male Sprague Dawley rats, weighing 200–250 g and aged 4–6 weeks, were used in this study. The rats were randomly divided into four groups as follows: The Control group (n = 8) consists of healthy rats without induced subarachnoid hemorrhage (SAH). The SAH group (n = 8) includes rats with induced SAH but no treatment administered. The Saline group (n = 8) comprises rats with induced SAH, followed by subcutaneous administration of 0.2 mL saline. Finally, the Anakinra group (n = 8) consists of rats with induced SAH, followed by subcutaneous administration of anakinra at a dose of 50 mg/kg twice daily [11].

This study was conducted in compliance with the ethical principles outlined in the Guide for the Care and Use of Laboratory Animals and was approved by the Institutional Animal Care and Use Committee (IACUC) of Düzce University. All procedures were performed in accordance with national and international guidelines to ensure animal welfare, and every effort was made to minimize animal suffering (Ethics Approval No: 2024/01/02; date: 16 January 2024).

### 2.2. Subarachnoid Hemorrhage Induction

The SAH model was established based on methods described in the literature. Rats in the experimental groups were anesthetized with a combination of ketamine (35 mg/kg) and xylazine (5 mg/kg) administered subcutaneously. Through a micro-surgical approach to the cisterna magna, 0.1 mL of cerebrospinal fluid (CSF) was aspirated and replaced with an equal volume of non-heparinized arterial blood [12].

The basilar artery was chosen for evaluation because it is one of the vessels most susceptible to vasospasm after SAH. In terms of anatomy and physiology, the basilar artery is a widely used reference vessel in SAH models and has a suitable structure for histopathologic examinations. Moreover, changes in the diameter of the basilar artery are considered an important indicator to determine the degree of vasospasm. Therefore, in our study, basilar artery lumen diameter was measured to assess the severity of vasospasm.

### 2.3. Biochemical Assessment

In this study, the time-dependent effects of subcutaneously (SC) administered anakinra on acute inflammation developing in a subarachnoid hemorrhage (SAH) model in rats were investigated. Anakinra was administered at a dose of 50 mg/kg to SAH model groups at different time points (3rd, 8th, and 14th day), and CRP, TNF-α, IL-1β, IL-6, and fibrinogen levels were measured to evaluate the time-dependent changes in inflammation. Measurements were performed in both cerebrospinal fluid (CSF) and blood serum to compare systemic proinflammatory effects. Blood samples (minimum volume: 0.5 mL) were collected via venipuncture into biochemistry tubes containing gel. The tubes were left at room temperature for 10 min and then centrifuged at 1800× *g* for 10 min. Separated serum samples were initially stored at −20 °C and subsequently kept at −80 °C until analysis. Levels of CRP, TNF-α, IL-1β, IL-6, and fibrinogen in the serum and CSF were measured using commercially available rat-specific enzyme immunoassay kits (Catalog Numbers: E0053Ra BTLab; E0764Ra BTLab; E0107Ra BTLab; E0135Ra BTLab; EA0056Ra BTLab, BTLab, Shanghai, China). Spectrophotometric readings at 460 nm OD absorbance were performed using a BIOTEK ELX800 microplate reader (Thermo Fisher Scientific, Waltham, MA, USA) in accordance with the kit protocols. The obtained data were analyzed using a two-point calibration curve and standard reagent barcodes. Curvexpert software (version 2.7.3, Hyams Development) was utilized for these analyses. To ensure the reliability of our measurements, all ELISA assays were performed in triplicate, and internal quality control samples were included in each run. The test kits used in this study are commercially validated and have been previously utilized in similar experimental models. This approach allowed for the evaluation of changes in acute inflammatory findings, which began to manifest with the migration of blood cells to the hemorrhage site at the 3rd hour after SAH induction, using samples taken at various time points following anakinra administration [13].

### 2.4. Histopathological Examination of the Basilar Artery

For histopathological examination, four-micron sections were obtained from paraffin-embedded blocks. These sections were mounted on standard glass slides and then incubated in an oven at 75 °C for 40 min to remove the paraffin. After deparaffinization, the sections were placed in xylene twice, for 10 min each, to remove the residual paraffin. This was followed by a series of graded ethanol washes (100%, 90%, 80%, and 70%) with 10 dips each to rehydrate the tissue. Next, the sections were stained with hematoxylin for 3 min to visualize the nuclei. They were then rinsed thoroughly in running water. To achieve proper staining intensity, the sections were briefly dipped twice in acid alcohol followed by another rinse in running water. A brief double dip in ammonia water with a final rinse ensured optimal staining conditions. Finally, the sections were washed in 70% ethanol ten times. For cytoplasmic staining, the sections were immersed in an alcohol-based eosin solution for 40 s, followed by another water rinse. They were then dehydrated through a series of graded ethanol washes (70%, 80%, and 96%) with ten dips each. After a 10 min clearing step in xylene, the slides were heated in an oven at 75 °C for 2 min to complete the dehydration process. The stained slides were then mounted with a coverslip using entellan, a mounting medium. The basilar artery sections were photographed at 400× magnification using an AxioCam Zeiss digital camera attached to an Olympus Cx41 microscope. Using ImageJ software (version 1.8.0), the thickness of the basilar artery wall was measured from five different regions of the section using the diameter tool. The software automatically calculated the vessel diameter based on the thickness measurements [14].

### 2.5. Immunohistochemistry

**Figure 1:**
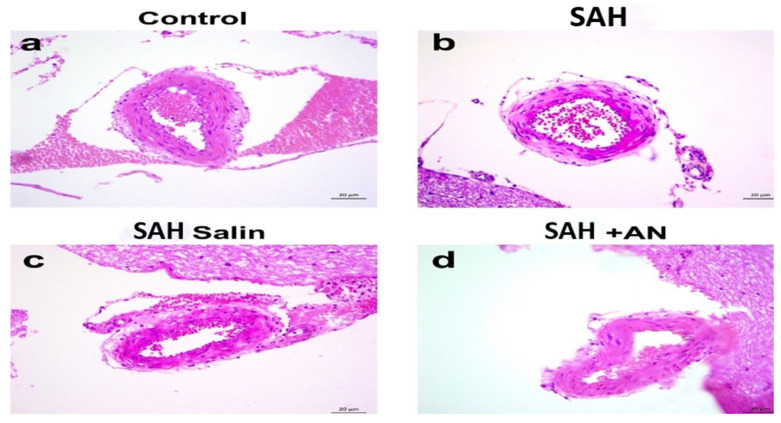
Representative photographs of hematoxylin and eosin staining. Objective magnification is 40× and scale bar is 20 µm.

**Figure 2:**
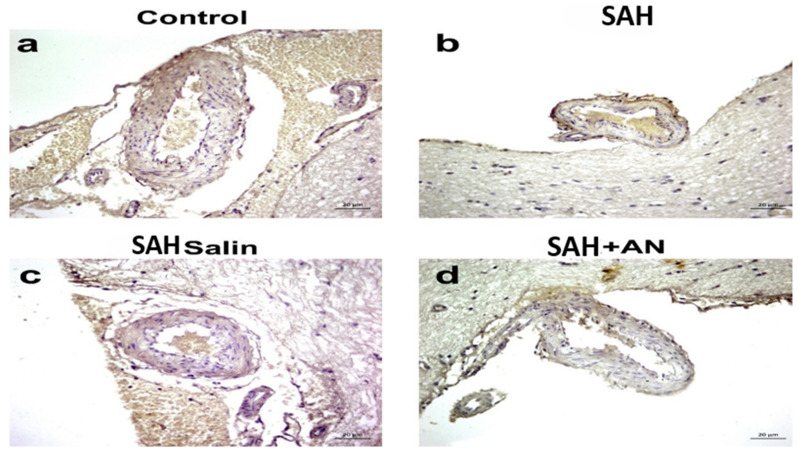
Representative photographs of cleaved Caspase-3 immunohistochemistry results. Objective magnification is 40× and scale bar is 20 µm.

Four-micron sections were mounted on poly-L-lysine-coated slides to improve tissue adherence. Following deparaffinization and rehydration, antigen retrieval was performed using citrate buffer (pH 6.0) at high temperature. Sections were then boiled in a 750-watt microwave oven for 7 min, followed by cooling and washing with phosphate-buffered saline (PBS). Endogenous peroxidase activity was blocked with 3% hydrogen peroxide for 15 min. To minimize non-specific antibody binding, the sections were incubated with a protein blocking solution (UltraV Block) for 7 min. Cleaved Caspase-3, a marker of apoptosis (programmed cell death), was detected using a rabbit polyclonal primary antibody (Cell Signaling, #9664) diluted at 1:200. Incubation occurred overnight at 4 °C to allow for specific antibody–antigen interactions. The next day, the unbound primary antibody was removed by washing with PBS. A biotinylated secondary antibody was applied for 30 min, followed by further PBS washes. Finally, a peroxidase-labeled streptavidin conjugate was incubated for 15 min to link the secondary antibody to the visualization system. Immunohistochemical staining was achieved by incubating the sections with diaminobenzidine (DAB). The sections were then counterstained with hematoxylin to visualize nuclei and dehydrated before coverslipping with entellan. Photomicrographs of the immunostained sections were captured at 40× magnification using an AxioCam Zeiss digital camera attached to an Olympus Cx41 microscope. Ten images were obtained from each section. ImageJ software (version 1.8.0) was used to quantify the immunostaining intensity in each image. The percentage of the area occupied by brown DAB precipitate (representing Caspase-3 expression) was calculated relative to the total tissue area in each photograph [14,15].( Figure 1 and Figure 2).

### 2.6. Statistical Analysis

Statistical analyses were performed with the IBM SPSS v.22 (IBM Corp. Released 2013. IBM SPSS Statistics for Windows, Version 22.0. Armonk, NY, USA: IBM Corp.) package. The distribution of data was examined with the Shapiro–Wilk test, and the variance homogeneity was analyzed with the Levene test. Repeated measures of ANOVA followed by Tukey HSD and Bonferroni post hoc tests were used for group and measurement period comparisons. The data were summarized as the mean and standard deviation, and the statistical significance level was considered to be 0.05.

## 3. Results

According to Table 1, the biochemical and histopathological parameters show statistically significant differences among the experimental groups. Serum CRP levels were significantly higher in the subarachnoid hemorrhage (SAH) group compared to the Control group on days 3, 7, and 10, reflecting an acute inflammatory response. In the Saline group, CRP levels were slightly lower than in the SAH group but remained elevated compared to the Control group. In contrast, the Anakinra group demonstrated a reduction in CRP levels over time, approaching levels similar to the Control group by day 10.

Serum TNF-α levels were consistently higher in the SAH and Saline groups than in the Control group across all time points, indicating systemic inflammation. The Anakinra group showed a marked reduction in TNF-α levels compared to the SAH and Saline groups, suggesting the anti-inflammatory effects of anakinra.

Similarly, IL-1 and IL-6 levels were significantly elevated in the SAH and Saline groups compared to the Control group. However, the Anakinra group exhibited a notable decrease in these cytokines, particularly by day 10, when the levels were comparable to those of the Control group.

Fibrinogen levels followed a similar trend, with the highest levels observed in the SAH and Saline groups, reflecting a proinflammatory and pro-coagulant state. Anakinra treatment reduced fibrinogen levels over time, bringing them closer to Control group levels by day 10.

In cerebrospinal fluid (CSF), CRP, TNF-α, IL-1, and IL-6 levels mirrored the patterns seen in serum, with the highest levels in the SAH and Saline groups. Anakinra treatment significantly reduced these markers, demonstrating its potential to mitigate central nervous system inflammation.

**Figure 3:**
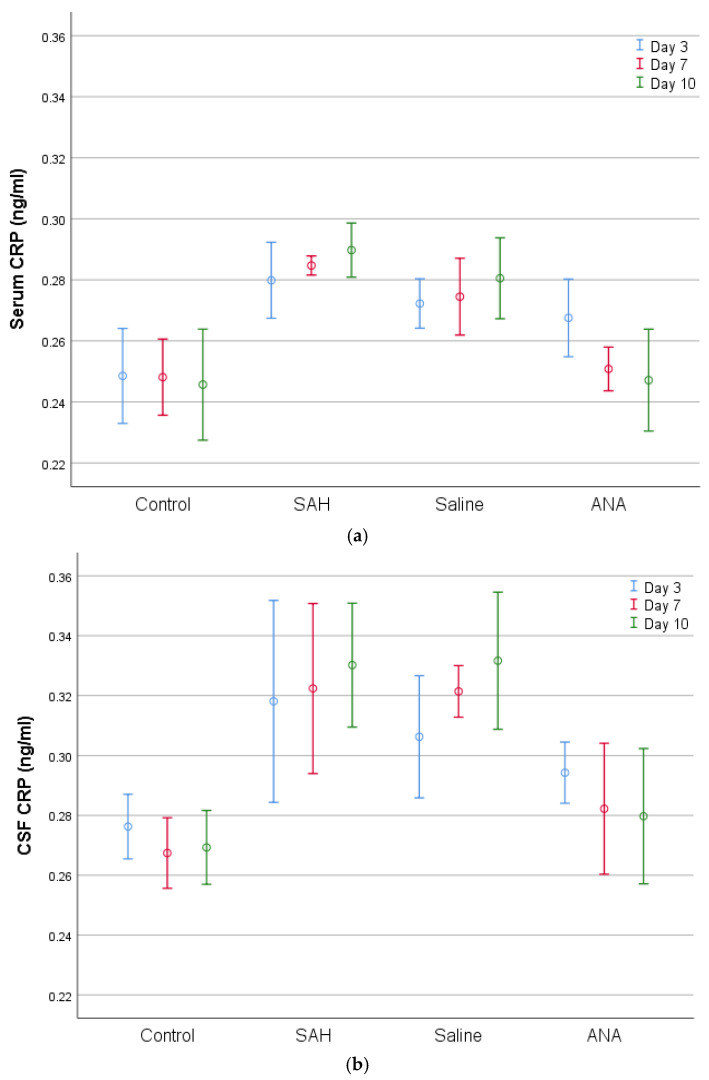
A comparison of (**a**) serum and (**b**) CSF CRP levels between groups. CRP values, which were statistically significantly increased in both serum and CSF with SAH compared to the Control group, decreased in the ANA treatment group. No significant difference was seen between the SAH and Saline groups in either serum or in CSF for the entire period of the experiment, and both groups showed significantly higher values compared to the Control group for all three periods of the experiment. While the decrease over the 3-day treatment period in the ANA group was not significant compared to the SAH and Saline groups, the levels of decrease were statistically significant over the 7-day and 10-day treatment periods both in serum and CSF. However, there was no statistically significant difference between the 3-day, 7-day, and 10-day treatment periods in the ANA group, although the CRP values continued to decrease over the treatment period and the lowest value that was closest to that of the Control group was detected on day 10. SAH: subarachnoid hemorrhage; ANA: anakinra; CSF: cerebrospinal fluid; CRP: C-reactive protein.

**Figure 4:**
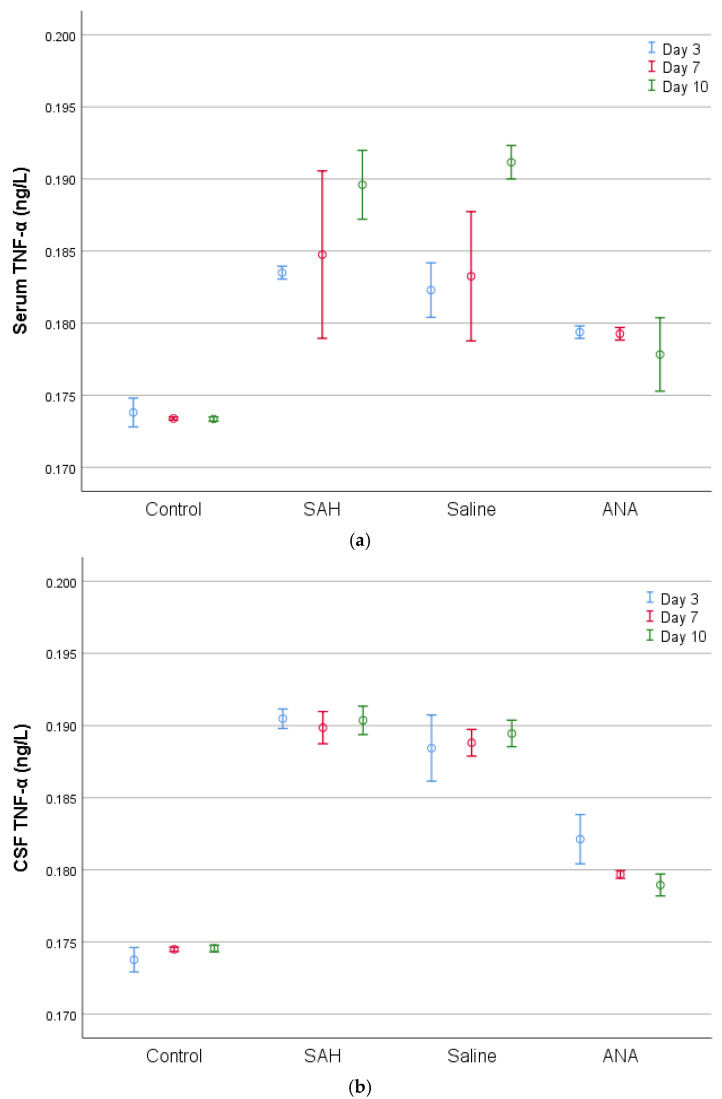
A comparison of (**a**) serum and (**b**) CSF TNF-α levels between groups. TNF-α values, which were statistically significantly increased in both serum and CSF with SAH compared to the Control group, decreased in the ANA treatment group. No significant difference was seen between the SAH and Saline groups in either serum or in CSF for the entire period of the experiment, and both groups showed significantly higher values compared to the Control group for all three periods of the experiment. While the decrease over the 3-day treatment period in the ANA group was significant compared to the SAH and Saline groups in both serum and CSF, the decrease over the 7-day treatment period did not show a significant difference, although the measured levels of TNF-α in serum were similar. Again, the level of decrease in the ANA group was statistically significant over the 10-day treatment period in both serum and CSF. While there was no statistically significant difference in the ANA group among the 3-day, 7-day, and 10-day values in serum, a significant change was observed in CSF, revealing significantly lower levels of TNF-α for the 7-day and 10-day periods in comparison to the 3-day period. SAH: subarachnoid hemorrhage; ANA: anakinra; CSF: cerebrospinal fluid; TNF-α: tumor necrosis factor-alpha.

**Figure 5:**
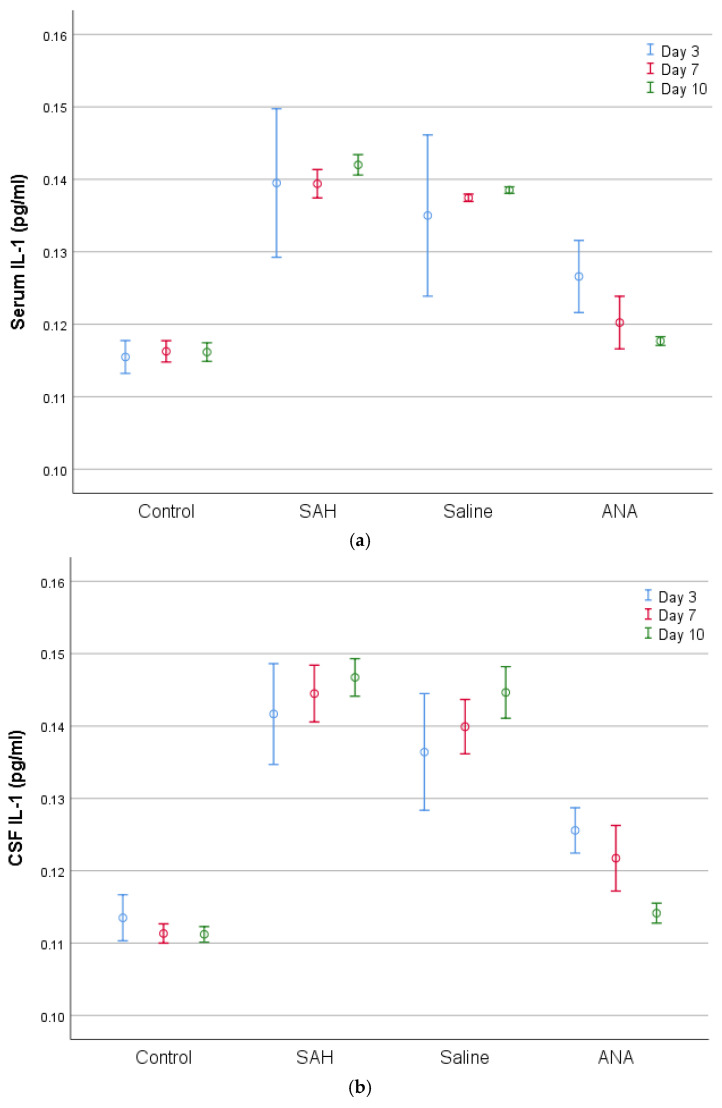
A comparison of (**a**) serum and (**b**) CSF IL-1 levels between groups. IL-1 values, which were statistically significantly increased in both serum and CSF with SAH compared to the Control group, decreased in the ANA treatment group. No significant difference was seen between the SAH and Saline groups in either serum or in CSF for the entire period of the experiment, and both groups showed significantly higher values compared to the Control group for all three periods of the experiment. The levels of decrease in the ANA group for all three treatment periods were statistically significant both in serum and CSF when compared to the SAH and Saline groups. On the other hand, a significant change was observed among the 3-day, 7-day, and 10-day values in both serum and CSF, revealing significantly lower levels of IL-1 for the 10-day period in comparison to the 3-day and 7-day periods. SAH: subarachnoid hemorrhage; ANA: anakinra; CSF: cerebrospinal fluid; IL-1: interleukin 1.

**Figure 6:**
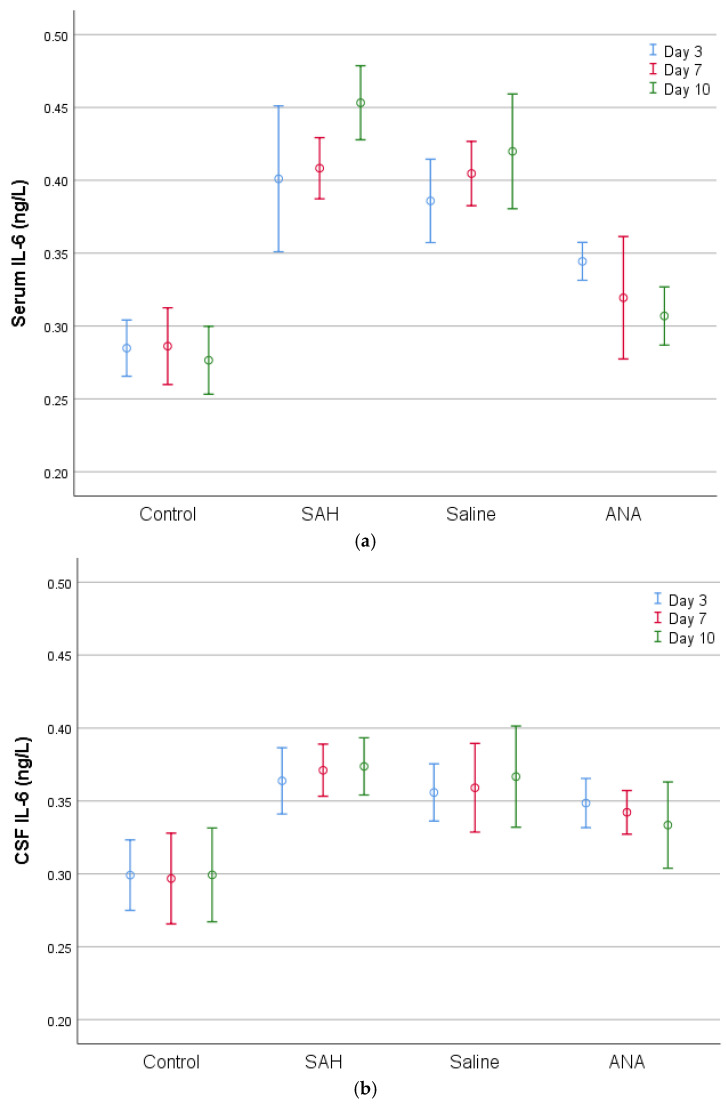
A comparison of (**a**) serum and (**b**) CSF IL-6 levels between groups. IL-6 values, which were statistically significantly increased in both serum and CSF with SAH compared to the Control group, decreased in the ANA treatment group. No significant difference was seen between the SAH and Saline groups in either serum or in CSF for the entire period of the experiment, and both groups showed significantly higher values compared to the Control group for all three periods of the experiment. While the decrease over the 3-day treatment period in the ANA group was not significant compared to the SAH and Saline groups, the levels of decrease were statistically significant over the 7-day and 10-day treatment periods in serum. In addition, there was no significant difference in CSF IL-6 levels between the ANA, SAH and Saline groups for any of the three treatment periods. While a significant change was observed in serum IL-6 in the ANA group among the 3-day, 7-day, and 10-day values, revealing significantly lower levels of IL-6 for the 7-day and 10-day periods in comparison to the 3-day period, there was no statistically significant difference in the ANA group among the 3-day, 7-day, and 10-day values in CSF. SAH: subarachnoid hemorrhage; ANA: anakinra; CSF: cerebrospinal fluid; IL-6: interleukin 6.

**Figure 7:**
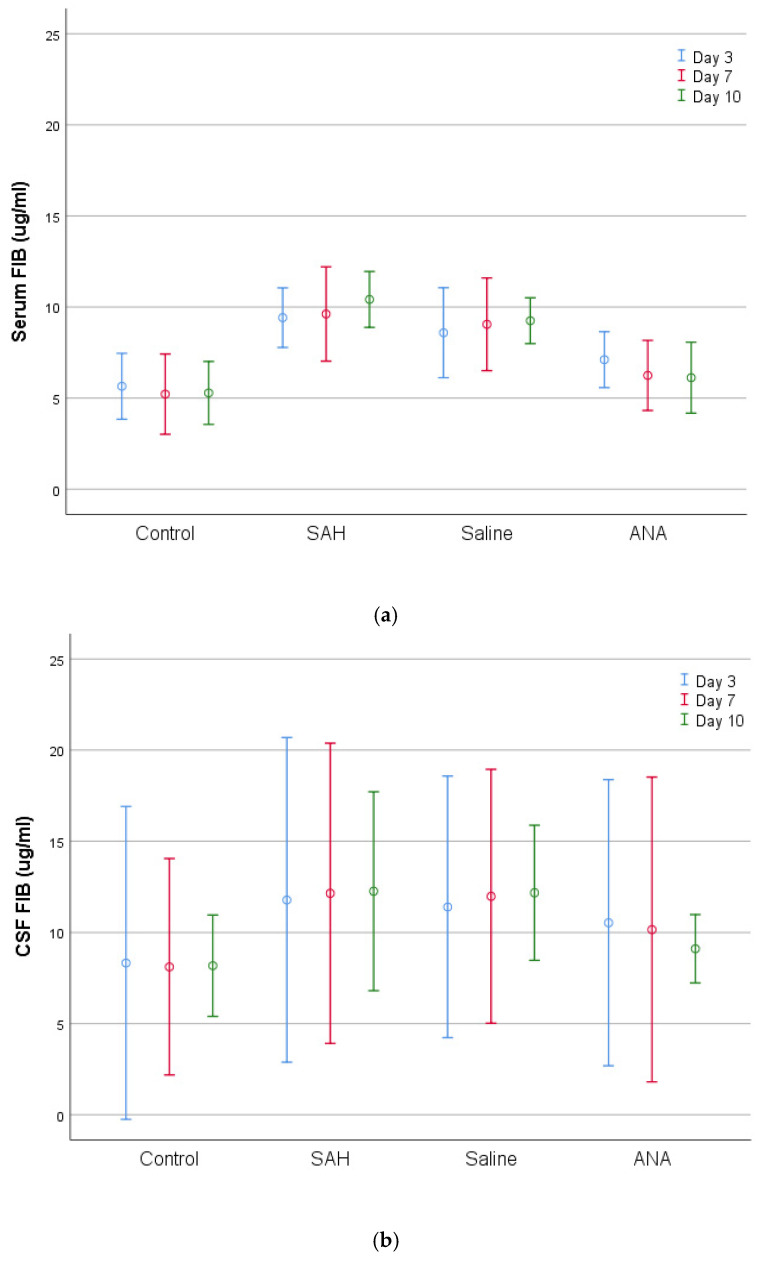
A comparison of (**a**) serum and (**b**) CSF FIB levels between groups. FIB values, which were statistically significantly increased in serum with SAH compared to Control group, remained at similar levels in the ANA treatment group. No significant difference was seen between the ANA, SAH, and Saline groups in either serum or CSF for all periods of the experiment, except for the 10-day period, where only the ANA group was significantly different from the SAH and Saline groups and similar to the Control group. However, there was no statistically significant difference between the 3-day, 7-day, and 10-day treatment periods in the ANA group, although the FIB values continued to decrease over the treatment period and the lowest value that was closest to that of the control group was detected on day 10. SAH: subarachnoid hemorrhage; ANA: anakinra; CSF: cerebrospinal fluid; FIB: fibrinogen.

**Figure 8:**
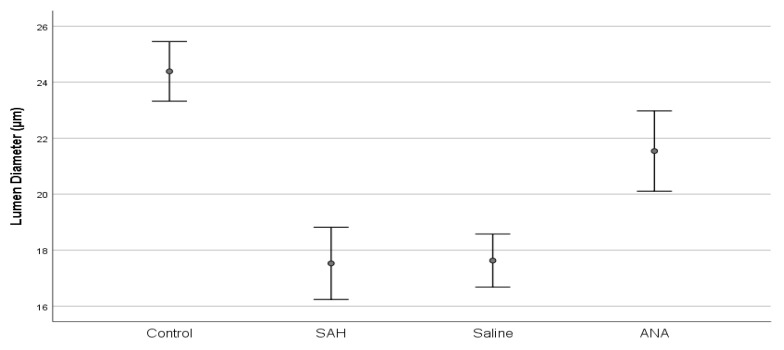
A comparison of lumen diameters between groups. Lumen diameter, which was statistically significantly decreased with SAH compared to the Control group, increased significantly in the ANA treatment group. No significant difference was seen between the SAH and Saline groups, and both groups showed significantly lower values compared to the Control group. SAH: subarachnoid hemorrhage; ANA: anakinra; *p* values of multiple comparisons: control vs. SAH (*p* < 0.001), control vs. saline (*p* < 0.001), control vs. ANA (*p* = 0.002), SAH vs. saline (*p* = 0.999), SAH vs. ANA (*p* < 0.001), and saline vs. ANA (*p* < 0.001).

**Figure 9:**
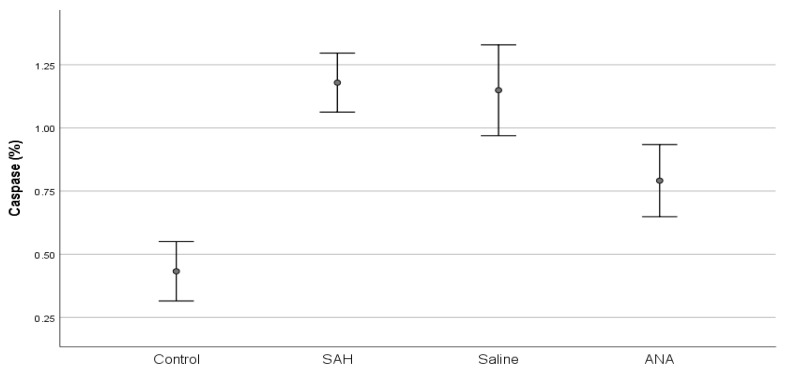
A comparison of Caspase levels between groups. Caspase level, which was statistically significantly increased with SAH compared to the Control group, decreased significantly in the ANA treatment group. No significant difference was seen between the SAH and Saline groups, and both groups showed significantly lower values compared to the Control group. SAH: subarachnoid hemorrhage; ANA: anakinra; *p* values of multiple comparisons: control vs. SAH (*p* < 0.001), control vs. saline (*p* < 0.001), control vs. ANA (*p* = 0.001), SAH vs. saline (*p* = 0.984), SAH vs. ANA (*p* < 0.001), and saline vs. ANA (*p* = 0.001).

Histopathologically, the basilar artery lumen diameter was significantly reduced in the SAH and Saline groups compared to the Control group, indicating vasospasm. Anakinra treatment partially restored the lumen diameter. Furthermore, Caspase-3 levels, indicative of apoptosis, were highest in the SAH and Saline groups but were significantly lower in the Anakinra group, suggesting its protective effects against apoptotic damage (Figure 3, Figure 4, Figure 5, Figure 6, Figure 7, Figure 8 and Figure 9).

## 4. Discussion

In this study, the effects of anakinra on inflammation, cerebral vasospasm, and apoptosis following subarachnoid hemorrhage (SAH) were evaluated, yielding significant findings consistent with the existing literature. Proinflammatory markers, such as CRP, TNF-α, IL-1, and IL-6, were found to be significantly elevated in the SAH group compared to the Control group, highlighting the intensity of the inflammatory response post-SAH. These results align with previous reports, which emphasize that elevated cytokine levels contribute to endothelial dysfunction and cerebral vasospasm [4,16,17]. In the Saline group, a slight reduction in these markers was observed compared to the SAH group. However, this reduction was limited, suggesting that saline administration alone is insufficient to adequately suppress inflammation. While saline has been reported to provide some stabilization in inflammatory responses [18], the substantial decrease observed in the Anakinra group reinforces its efficacy as an IL-1 receptor antagonist, targeting the key inflammatory pathways more effectively [19].

The role of proinflammatory cytokines in exacerbating the pathological consequences of SAH has been well documented. Elevated levels of IL-1 and TNF-α have been particularly associated with endothelial dysfunction and the disruption of the blood–brain barrier, which in turn facilitates secondary neuronal injury and vasospasm [20,21]. The significant reduction in these markers observed in the Anakinra group underscores the therapeutic potential of IL-1 inhibition in modulating these inflammatory cascades. Previous studies have shown that IL-1 plays a central role in the inflammatory cascade by stimulating the release of other proinflammatory cytokines, including TNF-α [4,13]. The observed decrease in TNF-α levels following anakinra administration may be attributed to the inhibition of IL-1-mediated inflammatory signaling. This finding is consistent with previous research indicating that IL-1 receptor antagonists can indirectly suppress TNF-α production, thereby attenuating the overall inflammatory response [4,13,20].

Similar studies, such as those by Kılıç et al. (2024) [4], have highlighted the broader anti-inflammatory and neuroprotective effects of IL-1 antagonists, supporting the findings of the current study. Previous research has explored anakinra in both human and animal SAH models, demonstrating its capacity to modulate inflammation and potentially improve clinical outcomes. However, the degree to which anakinra can mitigate cerebral vasospasm and apoptosis in SAH models has not been fully elucidated, making our findings a valuable addition to the existing literature [4,20,21].

Histopathological analyses demonstrated significant reductions in the basilar artery lumen diameter in the SAH and Saline groups, indicating the development of cerebral vasospasm. This observation is in line with the existing literature, which highlights vasospasm as a major complication of SAH driven by inflammation [22,23]. Although the Saline group showed minor alleviation of vasospasm compared to the SAH group, the Anakinra group exhibited significantly greater restoration of the basilar artery lumen diameter. This improvement underscores the importance of controlling inflammation in the management of vasospasm.

The histopathological findings from this study reinforce the critical interplay between inflammation and cerebral vasospasm. Partial restoration of the basilar artery lumen diameter in the Anakinra group is consistent with the hypothesis that inflammation is a primary driver of vasospasm [24,25]. While saline provided some stabilization of vasospasm parameters, the improvements were minimal and not statistically significant compared to the SAH group. This observation aligns with the literature indicating that saline, while often used as a neutral control, lacks active therapeutic properties against the complex inflammatory pathways involved in SAH-induced vasospasm [26].

Caspase-3 levels, an indicator of apoptosis, were elevated in both the SAH and Saline groups, suggesting ongoing cellular damage via apoptotic pathways. While saline treatment showed minimal effects in reducing apoptosis [27], the Anakinra group demonstrated a significant decrease in Caspase-3 expression. This finding supports the neuroprotective effects of anakinra, which aligns with prior studies highlighting its potential to mitigate apoptosis through inflammation suppression [4,28].

The reduction in Caspase-3 expression in the Anakinra group highlights the drug’s potential role in mitigating apoptosis, a key mechanism of neuronal loss following SAH. Previous research has demonstrated that the apoptotic cascade is strongly linked to inflammatory cytokines, particularly IL-1β and TNF-α, which are known to activate pro-apoptotic pathways in the central nervous system [26,29]. By attenuating IL-1 activity, anakinra may disrupt this cascade, reducing neuronal apoptosis and potentially improving long-term neurological outcomes. These findings align with prior research that highlights the dual benefits of targeting cytokine pathways, demonstrating both anti-inflammatory and anti-apoptotic effects [30,31].

This study has several limitations that should be acknowledged. First, it was conducted in an experimental animal model, which may not fully replicate the complexities of human SAH. Additionally, only short-term effects (3, 7, and 10 days) of anakinra were evaluated, leaving its long-term effects unknown. The relatively small sample size may also limit the statistical power of the findings. Furthermore, this study did not assess the impact of varying doses or combination therapies, which could provide valuable insights into optimizing treatment strategies. Moreover, while we evaluated the basilar artery lumen diameter as a marker of vasospasm, other important histopathological changes such as vascular necrosis, inflammatory cell infiltration, and intimal thickening were not systematically analyzed. This represents a limitation of our study, as a more comprehensive histological assessment could provide additional insights into the effects of SAH and anakinra treatment. Future studies should incorporate these parameters to better characterize vascular pathology following SAH. Future research involving larger sample sizes, long-term studies, and clinical trials is needed to further validate these findings.

## 5. Conclusions

In conclusion, this study demonstrates that anakinra effectively reduces inflammation, alleviates cerebral vasospasm, and mitigates apoptosis following SAH. By significantly lowering proinflammatory markers such as CRP, TNF-α, IL-1, and IL-6, restoring the basilar artery lumen diameter, and decreasing Caspase-3 expression, anakinra shows promise as a therapeutic agent in the management of SAH and its secondary complications. However, further studies are required to confirm its clinical applicability and explore its long-term effects and optimal dosing strategy.

## Figures and Tables

**Table 1:** Comparison of biochemical markers, histopathological parameters, and cytokine levels between experimental groups.

	Control (n = 8)	SAH (n = 8)	Saline (n = 8)	ANA (n = 8)	*p*
Serum CRP (ng/mL) 3-day 7-day 10-day	0.2485 ± 0.0186 ^a^ 0.2481 ± 0.0149 ^a^ 0.2457 ± 0.0218 ^a^	0.2799 ± 0.0149 ^b^ 0.2847 ± 0.0037 ^b^ 0.2898 ± 0.0106 ^b^	0.2722 ± 0.0097 ^b^ 0.2745 ± 0.0150 ^b^ 0.2805 ± 0.0159 ^b^	0.2675 ± 0.0152 ^ab^ 0.2508 ± 0.0086 ^a^ 0.2471 ± 0.0200 ^a^	**0.002** **<0.001** **<0.001**
	0.832	0.283	0.509	0.060	
Serum TNF-α (ng/L) 3-day 7-day 10-day	0.1738 ± 0.0012 ^a^ 0.1734 ± 0.0001 ^a^ 0.1733 ± 0.0002 ^a^	0.1835 ± 0.0005 ^c^ 0.1848 ± 0.0069 ^b^ 0.1896 ± 0.0029 ^c^ *	0.1823 ± 0.0023 ^c^ 0.1833 ± 0.0054 ^b^ 0.1912 ± 0.0014 ^c^ **	0.1794 ± 0.0005 ^b^ 0.1793 ± 0.0005 ^ab^ 0.1778 ± 0.0031 ^b^	**<0.001** **<0.001** **<0.001**
	0.342	**0.028**	**0.001**	0.216	
Serum IL-1 (pg/mL) 3-day 7-day 10-day	0.1155 ± 0.0027 ^a^ 0.1163 ± 0.0018 ^a^ 0.1162 ± 0.0015 ^a^	0.1395 ± 0.0123 ^b^ 0.1394 ± 0.0023 ^c^ 0.1420 ± 0.0017 ^c^	0.1350 ± 0.0133 ^b^ 0.1375 ± 0.0006 ^c^ 0.1385 ± 0.0005 ^b^	0.1266 ± 0.0060 ^ab^ 0.1202 ± 0.0043 ^b^ * 0.1177 ± 0.0007 ^a^ **	**<0.001** **<0.001** **<0.001**
	0.773	0.605	0.532	**<0.001**	
Serum IL-6 (ng/L) 3-day 7-day 10-day	0.2848 ± 0.0231 ^a^ 0.2862 ± 0.0315 ^a^ 0.2765 ± 0.0279 ^a^	0.4010 ± 0.0599 ^c^ 0.4083 ± 0.0251 ^b^ 0.4532 ± 0.0304 ^b^**	0.3859 ± 0.0342 ^bc^ 0.4047 ± 0.0264 ^b^ 0.4199 ± 0.0471 ^b^	0.3444 ± 0.0156 ^b^ 0.3194 ± 0.0503 ^a^ 0.3069 ± 0.0239 ^a^*	**<0.001** **<0.001** **<0.001**
	0.724	**0.008**	0.286	**0.001**	
Serum FIB (μg/mL) 3-day 7-day 10-day	5.6477 ± 2.1642 ^a^ 5.2180 ± 2.6341 ^a^ 5.2843 ± 2.0682 ^a^	9.4141 ± 1.9583 ^b^ 9.6166 ± 3.0962 ^b^ 10.4136 ± 1.8353 ^b^	8.5889 ± 2.9554 ^ab^ 9.0495 ± 3.0433 ^b^ 9.2485 ± 1.5037 ^b^	7.1111 ± 1.8366 ^ab^ 6.2432 ± 2.2994 ^ab^ 6.1212 ± 2.3290 ^a^	**0.013** **0.009** **<0.001**
	0.795	0.695	0.845	0.560	
CSF CRP (ng/mL) 3-day 7-day 10-day	0.2763 ± 0.0129 ^a^ 0.2674 ± 0.0141 ^a^ 0.2693 ± 0.0148 ^a^	0.3181 ± 0.0403 ^b^ 0.3224 ± 0.0339 ^b^ 0.3302 ± 0.0248 ^b^	0.3063 ± 0.0244 ^ab^ 0.3214 ± 0.0103 ^b^ 0.3316 ± 0.0274 ^b^	0.2943 ± 0.0122 ^ab^ 0.2822 ± 0.0262 ^a^ 0.2797 ± 0.0271 ^a^	**0.011** **<0.001** **<0.001**
	0.145	0.806	0.061	0.228	
CSF TNF-α (ng/L) 3-day 7-day 10-day	0.1738 ± 0.0010 ^a^ 0.1745 ± 0.0002 ^a^ 0.1745 ± 0.0003 ^a^	0.1905 ± 0.0008 ^c^ 0.1899 ± 0.0013 ^c^ 0.1904 ± 0.0012 ^c^	0.1884 ± 0.0027 ^c^ 0.1888 ± 0.0011 ^c^ 0.1895 ± 0.0011 ^c^	0.1821 ± 0.0020 ^b^ 0.1797 ± 0.0003 ^b^ * 0.1789 ± 0.0009 ^b^ *	**<0.001** **<0.001** **<0.001**
	0.074	0.589	0.528	**0.001**	
CSF IL-1 (pg/mL) 3-day 7-day 10-day	0.1135 ± 0.0038 ^a^ 0.1113 ± 0.0016 ^a^ 0.1112 ± 0.0013 ^a^	0.1417 ± 0.0083 ^c^ 0.1445 ± 0.0047 ^c^ 0.1467 ± 0.0031 ^b^	0.1364 ± 0.0096 ^c^ 0.1399 ± 0.0045 ^c^ 0.1446 ± 0.0043 ^b^	0.1256 ± 0.0038 ^b^ 0.1217 ± 0.0054 ^b^ 0.1141 ± 0.0017 ^a^ **	**<0.001** **<0.001** **<0.001**
	0.152	0.269	0.091	**<0.001**	
CSF IL-6 (ng/L) 3-day 7-day 10-day	0.2991 ± 0.0289 ^a^ 0.2968 ± 0.0372 ^a^ 0.2993 ± 0.0385 ^a^	0.3638 ± 0.0272 ^b^ 0.3711 ± 0.0214 ^b^ 0.3738 ± 0.0235 ^b^	0.3559 ± 0.0235 ^b^ 0.3591 ± 0.0364 ^b^ 0.3667 ± 0.0415 ^b^	0.3486 ± 0.0202 ^b^ 0.3422 ± 0.0179 ^b^ 0.3335 ± 0.0354 ^ab^	**<0.001** **<0.001** **0.001**
	0.823	0.559	0.798	0.510	
CSF FIB (μg/mL) 3-day 7-day 10-day	8.3261 ± 10.2630 8.1136 ± 7.0984 8.1809 ± 3.3281	11.7836 ± 10.6491 12.1461 ± 9.8459 12.2582 ± 6.5209	11.4019 ± 8.5792 11.9814 ± 8.3274 12.1738 ± 4.4240	10.5339 ± 9.3828 10.1589 ± 9.9960 9.1103 ± 2.2447	0.897 0.783 0.170
	0.972	0.918	0.861	0.862	
Lumen Diameter (µm)	24.3860 ± 1.2732 ^a^	17.5290 ± 1.5429 ^b^	17.6293 ± 1.1342 ^b^	21.5406 ± 1.7173 ^c^	**<0.001**
Caspase (%)	0.4325 ± 0.1407 ^a^	1.1792 ± 0.1399 ^b^	1.1492 ± 0.2152 ^b^	0.7913 ± 0.1708 ^c^	**<0.001**

SAH: subarachnoid hemorrhage, ANA: anakinra, TCZ: tocilizumab, CRP: C-reactive protein, TNF-α: tumor necrosis factor-alpha, IL: interleukin, FIB: fibrinogen, CSF: cerebrospinal fluid, a, b, c: different superscript letters denote significant differences between the groups according to the between-group post hoc test, *: symbols in different numbers indicate significant differences between the days measured according to the within-group post hoc test.

## Data Availability

The data that support the findings of this study are available from the corresponding author upon reasonable request.

## Abbreviations

ANA	anakinra
CRP	C-reactive protein
CSF	cerebrospinal fluid
FIB	fibrinogen
H&E	hematoxylin and eosin
IL	interleukin
SAH	subarachnoid hemorrhage
TNF-α	tumor necrosis factor-alpha
